# Supplementary material for: Cytomegalovirus in biliary atresia is associated with increased pretransplant death, but not decreased native liver survival
Source: Hepatol Commun. 2023 Jul 17;7(8):e0175. doi: 10.1097/HC9.0000000000000175 (PMC10351947; doi:10.1097/HC9.0000000000000175)
Supplement: Supplementary file 1 [file hc9-7-e0175-s001.docx]

Supplementary Table 1: Characteristics of participants with biliary atresia (BA) who died pre-liver transplant during the study, based on CMV status: CMV+ (A) and CMV- (B)

1A:

| **CMV+ Participant** | **Comorbidities** | **BA Related Complications** | **Age at HPE (days)** | **Jaundice cleared** | **Steroid Use** | **Pathology**  **(Ishak Stage)** | **Age at Transplant Listing** | **Age at Death** | **Cause of Death** |
| --- | --- | --- | --- | --- | --- | --- | --- | --- | --- |
| 1 | None | Varices | 73 | Yes (3 and 6 mo post-HPE) | Yes (3 mo post-HPE) | Stage 6 | N/A | 9.8 mo | Anoxic brain damage and hepatic coma |
| 2 | None | Ascites, failed HPE, varices, cholangitis, GI bleeding | 57 | Unknown | No | Unknown | 6 mo | 9.5 mo | Unknown |
| 3 | CHD, heterotaxy, polysplenia, situs inversus, midline liver | Jaundice, cirrhosis, FTT, failed HPE, sepsis | 66 | Unknown | No | Stage 3 | N/A | 5.4 mo | Respiratory failure from high output cardiac failure and hemorrhagic pulmonary edema, sepsis |
| 4 | CHD, heterotaxy, intestinal malrotation, right sided stomach, left sided liver, right sided spleen | None | 77 | Yes (3 mo post-HPE) | No | Stage 3 | N/A | 9.4 mo | Cardiac arrest |

1B:

| **CMV- Participant** | **Comorbidities** | **BA Related Complications** | **Age at HPE (days)** | **Jaundice cleared** | **Steroid Use** | **Pathology (Ishak Stage)** | **Age at Transplant Listing** | **Age at Death** | **Cause of Death** |
| --- | --- | --- | --- | --- | --- | --- | --- | --- | --- |
| 1 | CHD, omphalocele, ileal atresia, GU abnormalities, intestinal malrotation | FTT, failed HPE, coagulopathy, encephalopathy, GI bleed | 37 | No (3 mo post-HPE) | No | Stage 2 | N/A | 8.9 mo | Electrolyte abnormalities and liver failure |
| 2 | CHD, intestinal malrotation, polysplenia | Jaundice, cirrhosis, FTT, ascites, failed HPE, coagulopathy | 80 | No (3 mo post-HPE) | No | Stage 2 | 6 mo | 7.5 mo | Massive abdominal and pulmonary hemorrhage |
| 3 | None | FTT, ascites, failed HPE, coagulopathy, portal hypertension, cholangitis | 66 | Unknown | No | Stage 3 | 9 mo | 12.4 mo | Unknown cause |
| 4 | CHD, heterotaxy, duodenal/jejunal atresia, midline liver, right sided stomach, polysplenia | FTT, failed HPE, coagulopathy | 61 | Unknown | No | Unknown | N/A | 5.7 mo | Multisystem organ failure |
| 5 | CHD, TEF-esophageal atresia, vertebral and rib anomalies | FTT, ascites, cholangitis, failed HPE, coagulopathy, encephalopathy, metabolic bone disease | 33 | No (3 and 6 mo post-HPE) | Yes (6 months post-HPE) | Stage 3 | N/A | 9.5 mo | Respiratory failure |
| 6 | CHD, heterotaxy, anomalous portal vein, intestinal malrotation, midline liver, right sided stomach, situs inversus | Encephalopathy, hepatopulmonary syndrome, ascites | 44 | Yes (3 and 6 mo post-HPE) | Yes (2 yo) | Stage 2 | N/A | 42 mo | Respiratory failure |
| 7 | None | Ascites, coagulopathy, varices, GI bleed | 75 | Unknown | No | Stage 3 | N/A | 5.8 mo | Acute respiratory failure, varices, GI bleed |
| 8 | CHD, heterotaxy, intestinal malrotation, polysplenia | FTT, ascites, failed HPE, varices, sepsis | 58 | Yes (3 mo post-HPE) | No | Stage 4 | N/A | 11.4 mo | Acute respiratory distress syndrome |

CHD = congenital heart disease; FTT = failure to thrive; GU = genitourinary, N/A = not applicable
